# Fast and accurate clustering of noncoding RNAs using ensembles of sequence alignments and secondary structures

**DOI:** 10.1186/1471-2105-12-S1-S48

**Published:** 2011-02-15

**Authors:** Yutaka Saito, Kengo Sato, Yasubumi Sakakibara

**Affiliations:** 1 Department of Biosciences and Informatics, Keio University, 3-14-1 Hiyoshi, Kohoku-ku, Yokohama, Kanagawa 223-8522, Japan; 2 Graduate School of Frontier Sciences, University of Tokyo, 5-1-5 Kashiwanoha, Kashiwa, Chiba 277-8561, Japan; 3 Computational Biology Research Center (CBRC), National Institute of Advanced Industrial Science and Technology (AIST), 2-42 Aomi, Koto-ku, Tokyo 135-0064, Japan

## Abstract

**Background:**

Clustering of unannotated transcripts is an important task to identify novel families of noncoding RNAs (ncRNAs). Several hierarchical clustering methods have been developed using similarity measures based on the scores of structural alignment. However, the high computational cost of exact structural alignment requires these methods to employ approximate algorithms. Such heuristics degrade the quality of clustering results, especially when the similarity among family members is not detectable at the primary sequence level.

**Results:**

We describe a new similarity measure for the hierarchical clustering of ncRNAs. The idea is that the reliability of approximate algorithms can be improved by utilizing the information of suboptimal solutions in their dynamic programming frameworks. We approximate structural alignment in a more simplified manner than the existing methods. Instead, our method utilizes *all possible* sequence alignments and *all possible* secondary structures, whereas the existing methods only use *one optimal* sequence alignment and *one optimal* secondary structure. We demonstrate that this strategy can achieve the best balance between the computational cost and the quality of the clustering. In particular, our method can keep its high performance even when the sequence identity of family members is less than 60%.

**Conclusions:**

Our method enables fast and accurate clustering of ncRNAs. The software is available for download at http://bpla-kernel.dna.bio.keio.ac.jp/clustering/.

## Background

Noncoding RNAs (ncRNAs) serve a variety of cellular functions depending on their primary sequences and secondary structures [1]. A group of ncRNAs sharing sequence and structural homology is annotated as one family, and included in the database [2]. Recently, high-throughput transcriptome sequencing has uncovered tens of thousands of ncRNAs that lack significant homology to known families [3,4]. Thus, evaluating homology *among* these unannotated transcripts, that is, *clustering* has become an important task to identify novel ncRNA families [5,6].

Accurate clustering of ncRNAs needs a reliable similarity measure that takes into account primary sequences and secondary structures. Given a pair of sequences without known structures, the Sankoff algorithm [7] simultaneously predicts their sequence alignment and consensus secondary structure (*i.e.*, structural alignment); thus, the obtained alignment score can be a suitable choice for a similarity measure. However, the original Sankoff algorithm is too time-consuming to deal with an all-against-all comparison of many sequences required in clustering procedures. To address this problem, similarity measures based on the approximation of the Sankoff algorithm have been proposed, and shown to be applicable to hierarchical clustering [8-10]. Each method has its own heuristics to reduce the huge dynamic programming matrix used in the Sankoff algorithm. Will *et al*. [8] have developed LocARNA that precludes unsure secondary structures including low-probability base pairs. Torarinsson *et al*. [9] have developed FOLDALIGNM based on the FOLDALIGN program [11] that dynamically excludes low-scoring sequence alignments by means of length-dependant thresholds. Sato *et al*. [10] have developed Stem kernel that employs heuristics similar to LocARNA, but further precludes secondary structures including any bifurcation.

Although the approximate Sankoff-style algorithms have enabled similarity measures based on structural alignment, the quality of clustering results has not been so high. In the previous studies [6,8,12], resultant clusters in a hierarchical tree were quite unclear, requiring additional verification or manual inspection. This was partly because of the diversity within one ncRNA family. Most ncRNA families have only less than 60% identity at the primary sequence level [2], and cannot be correctly aligned without taking into account secondary structures [13]. The approximate Sankoff-style algorithms seemed to be degraded by discarding the secondary structures in the excluded portion of the dynamic programming matrix.

To improve the reliability of the approximate Sankoff-style algorithms, we focus attention on the information of suboptimal structural alignments. Among the existing methods, LocARNA and FOLDALIGN calculate the similarity based on the score of *one optimal* structural alignment. This means that these methods ignore the scores of suboptimal structural alignments, and only use *one optimal* sequence alignment and *one optimal* secondary structure. In contrast, Stem kernel sums up the scores of structural alignments allowed in the approximate Sankoff-style algorithm, incorporating a *subset of* sequence alignments and a *subset of* secondary structures. Thanks to this strategy, Stem kernel gives comparable clustering results to LocARNA, while employing the more reduced dynamic programming matrix. These observations suggest the possibility that we can design a more reliable similarity measure by utilizing *all possible* sequence alignments and *all possible* secondary structures. This is not trivial because if we naively try to incorporate all possible structural alignments, it will require the full-size dynamic programming matrix used in the original Sankoff algorithm with the prohibitive computational cost.

In this paper, we describe a new similarity measure for the hierarchical clustering of ncRNAs. We approximate the problem of structural alignment by the two separate problems: the prediction of sequence alignment, and the prediction of secondary structure for each sequence. For this purpose, the Sankoff algorithm for structural alignment is approximated by the combination of the Smith-Waterman (SW) algorithm [14] for sequence alignment, and the McCaskill algorithm [15] for secondary structures. The approximation allows to obtain all possible sequence alignments from the SW algorithm, and all possible secondary structures from the McCaskill algorithm, much faster than obtaining all possible structural alignments from the original Sankoff algorithm. We first describe a similarity measure using the scores of all possible sequence alignments between two RNAs. Then, we design a scoring function for these sequence alignments using all possible secondary structures of each of the two RNAs. We start from a scoring function that measures the similarity between two secondary structures using the state of base pairing at each position. The proposed scoring function is defined as an expectation of this scoring function over all possible secondary structures of each of the two RNAs.

We demonstrate that our method can achieve the best balance between the computational cost and the quality of the clustering among the existing methods. In particular, our method can keep its high performance even when the sequence identity of family members is less than 60%.

## Methods

In this section, we propose a new method for measuring the similarity between two RNA sequences without known structures. The proposed method is applied to the hierarchical clustering of ncRNAs with the weighted pair group method with averaging (WPGMA) algorithm. Given a set of sequences, we calculate an all-against-all similarity matrix using our method. Then, we derive the distance matrix by one minus the similarity, and obtain the cluster tree by the WPGMA algorithm.

The idea of our similarity measure is to approximate the Sankoff algorithm for structural alignment by the combination of the SW algorithm for sequence alignment, and the McCaskill algorithm for secondary structures. This approximation allows to utilize the ensembles of *all possible* sequence alignments and *all possible* secondary structures separately from each of the two algorithms. First, we describe a similarity measure using the scores of all possible sequence alignments between two RNAs. Next, we design a scoring function for these alignments using all possible secondary structures of each of the two RNAs.

### Ensemble of all possible sequence alignments

To measure the similarity between two RNAs, one common approach is to perform pairwise alignment, and to calculate its alignment score. The Sankoff algorithm simultaneously models sequence alignments and secondary structures, and is extremely time-consuming. Therefore, we first approximate the Sankoff algorithm by the SW algorithm that only models sequence alignments apart from secondary structures. Although this is a strong approximation, we attempt to improve the reliability by utilizing *all possible* sequence alignments rather than *one optimal* sequence alignment.

For an RNA sequence **x**, we denote its length by |**x**|. For each position 1 ≤ *i* ≤ |**x**| in **x**, we denote the nucleotide by *x_i_*∈ {A, C, G, U}.

For two sequences, **x** and **y**, let Π**_xy_** be the set of all possible sequence alignments in the SW algorithm. Let *π***_xy_** denote one particular sequence alignment in Π**_xy_**.

We calculate the similarity between **x** and **y** by accumulating the alignment score of *π***_xy_** over Π**_xy_**. For this purpose, we employ local alignment (LA) kernel [16] defined as follows:(1)

where *β* ≥ 0 is a parameter, and Score(*π***_xy_**) is the alignment score of *π***_xy_** under a given scoring scheme (gap penalties and match scores). In practice, we take the logarithm of LA kernel, and similarity values are normalized to range from 0 to 1:(2)

LA kernel (1) can be computed by the variant of the SW algorithm as follows:

Initialization:

**for*** i* ∈ {0,…, |**x**|} and *j* ∈ {0, …, |**y**|} **do**

*M*(*i*, 0) = *I_X_*(*i*, 0) = *I_Y_*(*i*, 0) = *T_X_*(*i*, 0) = *T_Y_*(*i*, 0) = 0

*M*(0, *j*) = *I_X_*(0, *j*) = *I_Y_*(0, *j*) = *T_X_*(0, *j*) = *T_Y_*(0, *j*) = 0

end for

Iteration:

**for*** i* ∈ {1,…,|**x**|} and *j* ∈ {1,…,|**y**|} **do**

*M*(*i*, *j*) = *e*^*βS*_xy_(*i,j*)^(1 + *I_X_*(*i* – 1, *j* – 1) + *I_Y_*(*i* – 1, *j* – 1) + *M*(*i* – 1, *j* – 1))

*I_X_*(*i*, *j*) = *e^βg^M*(*i* – 1, *j*) + *e^βd^I_X_*(*i* – 1, *j*)

*I_Y_*(*i*, *j*) = *e^βg^*(*M*(*i*, *j* – 1) + *I_X_*(*i*, *j* – 1)) + *e^βd^I_Y_*(*i*, *j* – 1)

*T_X_*(*i*, *j*) = *M*(*i* – 1, *j*) + *T_X_*(*i* – 1, *j*)

*T_Y_*(*i*, *j*) = *M*(*i*, *j* – 1) + *T_X_*(*i*, *j* – 1) + *T_Y_*(*i*, *j* – 1)

end for

Termination:

*K*(**x**, **y**) = 1 + *T_X_*(|**x**|, |**y**|) + *T_Y_*(|**x**|, |**y**|) + *M*(|**x**|, |**y**|)

where the parameters *g* and *d* are the penalties for gap opening and gap extension, respectively, and *S***_xy_**(*i*, *j*) is a scoring function for matching the *i*-th position in **x** and the *j-th* position in **y**. The design of *S***_xy_**(*i*, *j*) impacts the performance of the resulting similarity measure, and will be described later.

At this point, we note that our method can take into account all possible sequence alignments in *O*(|**x**||**y**|) time. If we use the exact Sankoff algorithm instead, it takes prohibitive *O*(|**x**|^3^|**y**|^3^) time, which is not practical. In the case of the approximate Sankoff-style algorithms employed in the existing methods, all possible sequence alignments cannot be incorporated to the reduced dynamic programming matrix. Therefore, LA kernel based on the SW algorithm is an efficient way to deal with the ensemble of all possible sequence alignments.

### Ensemble of all possible secondary structures

To design a scoring function *S***_xy_**(*i*, *j*) for LA kernel, we need secondary structures of **x** and **y**. As mentioned above, the Sankoff algorithm models secondary structures simultaneously with sequence alignments which we have already modeled by the SW algorithm. Therefore, we next employ the McCaskill algorithm that only models secondary structures apart from sequence alignments. Although this is an additional approximation, we attempt to improve the reliability by utilizing *all possible* secondary structures rather than *one optimal* secondary structure.

For an RNA sequence *x*, let Θ**_x_** be the set of all possible secondary structures. Let *θ***_x_** denote one particular secondary structure in Θ**_x_**. We represent a secondary structure as a set of binary variables *θ***_x_** = {*θ***_x_**(*i*, *j*)}_1≤_*_i_*_<_*_j_*_≤|_**_x_**_|_, where *θ***_x_**(*i*, *j*) = 1 means that the *i*-th position and the *j-th* position in **x** form a base pair. For each position 1 ≤ *i* ≤ |**x**| in **x**, we represent the state of base-pairing using three kinds of binary variable: *L***_x_**(*i*) = ∑ *_j:j_*_>_*_i_ θ***_x_**(*i*, *j*) = 1 means that a base pair is formed with one of the downstream positions; *R***_x_**(*i*) = ∑ *_j_*_:_*_j_*_<_*_i_ θ***_x_**(*j*, *i*) = 1 means that a base pair is formed with one of the upstream positions; and *U***_x_**(*i*) = 1 – *L***_x_**(*i*) – *R***_x_**(*i*) = 1 means that the position is unpaired. Given a fixed pair of secondary structures, *θ***_x_** and *θ***_y_**, we can measure the similarity between the *i*-th position in **x** and the *j-th* position in **y** using their state of base pairing:

*W***_xy_**(*i*, *j*|*θ***_x_**, *θ***_y_**) = *α* (*L***_x_**(*i*)*L***_y_**(*j*) + *R***_x_**(*i*)*R***_y_**(*j*)) + *s*(*x_i_*, *y_j_* ) *U***_x_**(*i*)*U***_y_**(*j*), (3)

where *α* ≥ 0 is a weight parameter for structural similarity, and *s*(*x_i_*, *y_j_*) is a substitution matrix for RNA sequences like the RIBOSUM 85-60 matrix [17]. This scoring function takes a non-zero value in three different cases: it takes *α* when both of the two positions form a base pair with one of their downstream positions, respectively; it takes *α* when both of the two positions form a base pair with one of their upstream positions, respectively; and it takes *s*(*x_i_*, *y_j_*) when both of the two positions are unpaired.

The McCaskill algorithm defines a probability distribution *P*(*θ***_x_**|**x**) over Θ**_x_**. The binary variables *θ***_x_**(*i*, *j*) and {*L***_x_**(*i*), *R***_x_**(*i*), *U***_x_**(*i*)} are converted to the probabilities by taking the expectation over Θ**_x_**. For *θ***_x_**(*i*, *j*), we obtain a base-pairing probability *P***_x_**(*i*, *j*) that the *i*-th and the *j-th* positions form a base pair:

For {*L***_x_**(*i*), *R***_x_**(*i*), *U***_x_**(*i*)}, we obtain three kinds of probability that the *i*-th position is paired with one of the downstream/upstream positions, or unpaired, respectively:

We design a scoring function *S***_xy_**(*i*, *j*) by taking the expectation of (3) over Θ**_x_** and Θ**_y_**:(4)

The proposed method is obtained by combining the normalized LA kernel (2) with the scoring function (4).

It should be noted that our method can take into account all possible secondary structures in *O*(|**x**|^3^ + |**y**|^3^) time, thanks to the McCaskill algorithm. Just as in all possible sequence alignments, the exact Sankoff algorithm results in *O*(|**x**|^3^|**y**|^3^) time, and the existing methods cannot incorporate all possible secondary structures. Our method requires *O*(|**x**||**y**|) + *O*(|**x**|^3^ + |**y**|^3^) time in total, which is more efficient than the exact Sankoff algorithm. Therefore, our strategy that combines the SW algorithm and the McCaskil algorithm allows to utilize the ensemble information with the reasonable computational cost.

### Variations of the proposed method

The scoring function (4) proposed in this study is similar to the scoring function used in BPLA kernel [18,19]. BPLA kernel is a prediction method that we previously developed for detecting new members of known ncRNA families. Although BPLA kernel was not applied to clustering problems in our previous study, we here clarify its relation to the proposed method. The scoring function used in BPLA kernel is defined as follows:(5)

where  ,  , and . Therefore, the scoring function (5) can be regarded as a variation of the proposed scoring function (4) with the additional coefficients *C^L^*, *C^R^*, and *C^U^*. These coefficients take large values when the probabilities  and  are small. That is, BPLA kernel emphasizes the contribution of low-probability (unsure) secondary structures compared to the proposed method. In the next section, we experimentally verify this theoretical implication; the proposed method outperforms BPLA kernel.

Because of the resemblance between the scoring functions, (4) and (5), we set the parameters of the proposed method as used in BPLA kernel: *α* = 1.0, *β* = 0.1, *g* = –27, and *d* = –0.1

## Results and discussion

In this section, we examine the performance of the proposed method in the hierarchical clustering of ncRNAs.

### Dataset and experimental system

We compared our method with the state-of-the-art methods developed for the hierarchical clustering of ncRNAs: LocARNA v1.5.2 [8], FOLDALIGN v2.1.1 [11], and Stem kernel v216c [10]. We also performed the experiments with CLUSTALW v1.83 [20] and LA kernel by setting  in our method (4).

We can summarize our method and the existing methods as follows. Our method utilizes *all possible* sequence alignments and *all possible* secondary structures. LocARNA and FOLDALIGN only use *one optimal* sequence alignment and *one optimal* secondary structures. Stem kernel utilizes a *subset of* all possible sequence alignments and a *subset of* all possible secondary structures. CLUSTALW and LA kernel ignore secondary structures; CLUSTALW only uses *one optimal* sequence alignment, while LA kernel utilizes *all possible* sequence alignments.

We created a dataset as summarized in Table 1. This dataset was collected from the BRAliBASE benchmark v2.1 [13], which includes multiple alignments of a broad range of ncRNA families established in the Rfam database [2]. We treated each multiple alignment as a reference cluster, and each ncRNA sequence in a multiple alignment as a member sequence. The reference clusters were divided into four categories according to their sequence identity: 20–39%, 40–59%, 60–79%, and 80–99%. We sampled the dataset ten times from the BRAliBASE benchmark, and evaluated the average performance.

**Table 1:** Summary of the dataset.

	20–39%	40–59%	60–79%	80–99%
#clusters	13	21	34	36
#members	3.2	5.0	3.8	4.6
Length	138	130	111	102

#clusters: number of reference clusters; each reference cluster represents a different ncRNA family.

#members: average number of member sequences per reference cluster. Length: average length of sequences over all reference clusters. The dataset is divided by the sequence identity in a reference cluster.

We produced three versions of dataset. First, we used ncRNA sequences without modification, and named them the “normal” dataset. Second, we concatenated random sequences to both ends of ncRNA sequences, and named them the “plus flanking regions” dataset. This dataset was intended to simulate the situation where we do not know the exact boundaries of unannotated transcripts. A random sequence was generated from a ncRNA sequence so that it had the quarter length and the same dinucleotide contents. Third, we added false reference clusters, each of which contains one random sequence, and named them the “plus unrelated sequences” dataset. This dataset was intended to simulate the situation where non-functional ncRNAs arise from transcriptional noises. Therefore, we evaluated whether a false reference cluster could be a resultant cluster with a single member. We used a quarter number of false reference clusters compared to true reference clusters. A random sequence was generated from a ncRNA sequence so that it had the same length and the same dinucleotide contents.

We evaluated the overall quality of the cluster tree by the ROC analysis proposed in [8]. (Note that we can obtain different resultant clusters from a cluster tree depending on a distance threshold to cut the branches.) Given a distance threshold, the number of true positives (*TP*) was defined as the number of sequence pairs that belong to the same reference cluster and are correctly assigned to the same resultant cluster. Analogously, the numbers of false positives (*FP*), true negatives (*TN*), and false negatives (*FN*) are defined, respectively, by counting the pairs from different reference clusters but the same resultant cluster, the pairs from different reference clusters and different resultant clusters, and the pairs from the same reference cluster but different resultant clusters. The ROC analysis was performed by plotting true positive rates *TP/*(*TP* + *FN*) versus false positive rates *FP/*(*TN* + *FP*) for different distance thresholds. The quality of the clustering was measured by the area under the ROC curve (AUC). We measured the total time for computing similarity matrices on a 2.53 GHz Intel Xeon processor.

### Quality of the clustering

**Figure 1:**
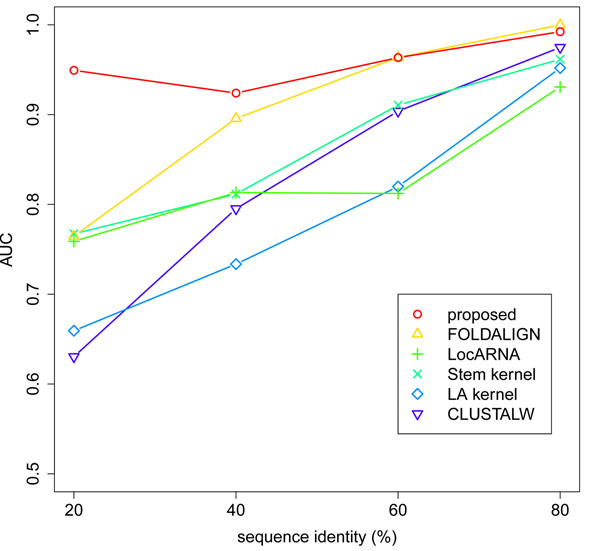
**Quality of the clustering for the “normal” dataset.** For each sequence identity range, the overall quality of the cluster tree is evaluated by the AUC.

We first examined the quality of the clustering for the “normal” dataset (Figure 1). Our method achieved the better or comparable AUC to the existing methods in all the range of sequence identity. The accuracy of our method was especially remarkable in the sequence identity range below 60%, where the existing methods resulted in low AUC. This means that our method successfully grouped diverse member sequences in each reference cluster by detecting their remote homology.

Our results can be attributed to the design of each method. The AUC of CLUSTALW and LA kernel, which ignore secondary structures and only use sequence alignments, drastically fell down as the sequence identity decreased. LocARNA, FOLDALIGN, and Stem kernel, which consider secondary structures, kept the AUC relatively moderate in the low sequence identity range. However, their accuracy was still limited when the sequence identity was extremely low (20–39%) because these methods only use *one optimal* secondary structure or a *subset of* secondary structures. Our method, which utilizes *all possible* sequence alignments and *all possible* secondary structures, achieved the sufficiently high AUC in this region. These results suggest that our design of the similarity measure is effective for identifying a broad range of ncRNA families.

**Figure 2:**
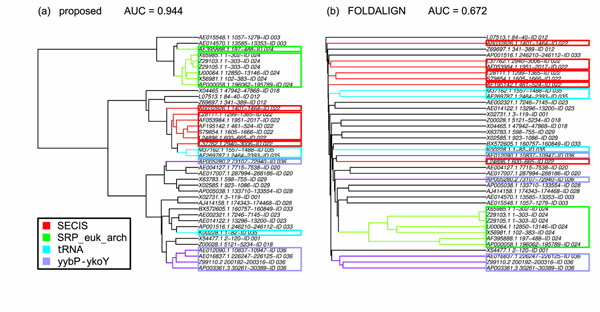
**Comparison of the cluster trees between the proposed method and FOLDALIGN.** For the sequence identity range of 20–39% in the “normal” dataset, an example of the cluster tree is shown with its AUC. In the leaf nodes, the strings such as “XXX-ID YYY” mean that the sequence XXX belongs to the reference cluster YYY. The four reference clusters that have more than two member sequences are colored, and their corresponding ncRNA families are noted.

Figure 2 compares an example of the cluster tree between our method and FOLDALIGN in the sequence identity rage of 20–39%. As indicated by AUC, our method produced the more accurate cluster tree than FOLDALIGN, and reconstructed ncRNA families as compact clusters. Although the cluster tree of FOLDALIGN was largely consistent with the references in terms of its topology, boundaries of resultant clusters were quite unclear. In the actual application of hierarchical clustering, we need to choose a proper distance threshold for extracting clusters from a given tree. In this sense, the cluster tree of FOLDALIGN was not sufficient for the practical use. In fact, the previous studies that employed clustering approaches required manual inspection to compensate for ambiguous cluster trees [6,8,12]. The cluster tree of our method was much more clear and easier to interpret than the existing methods. These results suggest that our method can reduce human labor costs of clustering approaches, and help to identify novel ncRNAs families.

**Figure 3:**
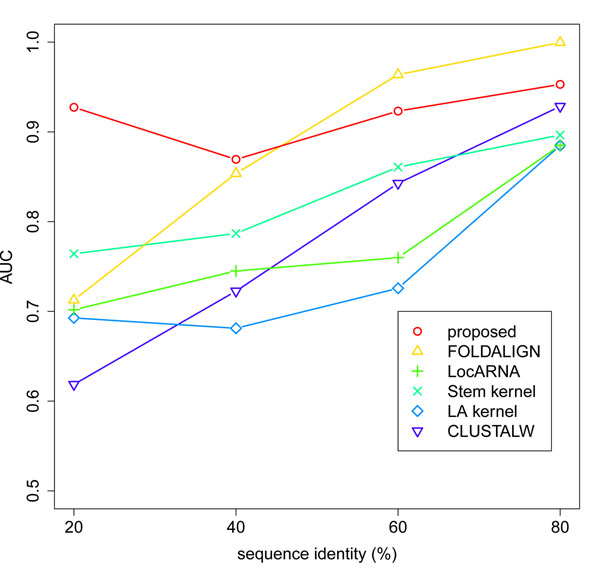
**Quality of the clustering for the “plus flanking regions” dataset.** For each sequence identity range, the overall quality of the cluster tree is evaluated by the AUC.

**Figure 4:**
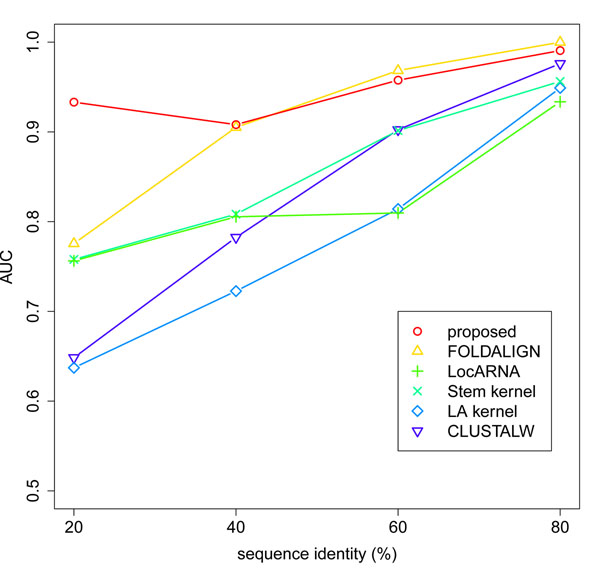
**Quality of the clustering for the “plus unrelated sequences” dataset.** For each sequence identity range, the overall quality of the cluster tree is evaluated by the AUC.

Next, we evaluated the quality of the clustering for the “plus flanking regions” dataset (Figure 3), and the “plus unrelated sequences” dataset (Figure 4). In both cases, we observed the same tendency as in the results for the “normal” dataset (Figure 1). Our method kept high accuracy in all the range of sequence identity, and achieved the best AUC in the sequence identity range below 60%. These results further support the effectiveness of our method in the practical situations that involve flanking regions and unrelated sequences.

### Differences in the variations of the proposed method

As described in Methods, the proposed method has the theoretical advantage compared to BPLA kernel, which can be regarded as a variation of our method. To verify this point experimentally, we compare the proposed method and BPLA kernel using the scoring functions (4) and (5), respectively.

**Figure 5:**
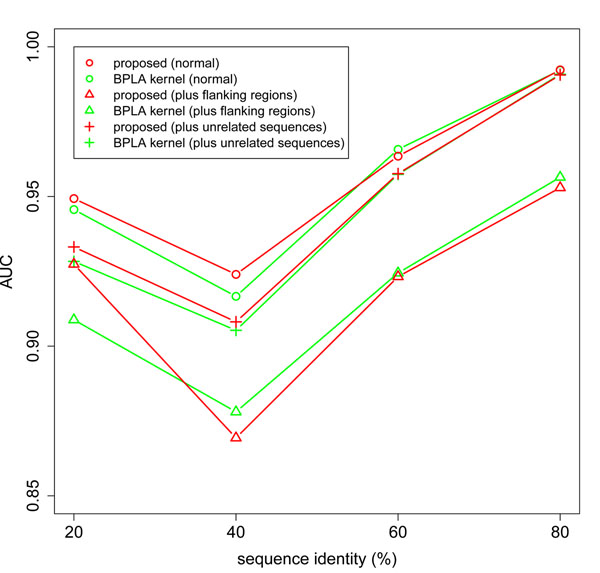
**Differences in the variations of the proposed method.** The proposed method is compared to BPLA kernel using three versions of the dataset. Note that BPLA kernel can be regarded as a variation of the proposed method.

Figure 5 presents the experimental results. The proposed method achieved the slightly better AUC in the sequence identity range below 60%. These results are consistent with the fact that BPLA kernel emphasizes the contribution of unsure secondary structures compared to the proposed method. The proposed scoring function (4) has the theoretical justification as the expectation of the primitive scoring function (3) over all possible secondary structures. Our results provide an experimental verification of the superiority of the proposed scoring function.

### Computational cost

Finally, we evaluated the computational cost of the similarity measures using three version of the dataset (Table 2). Our method was faster than LocARNA and FOLDALIGN by several orders of magnitude, and achieved the comparable computational cost to Stem kernel. Considering the high accuracy of our method (Figures 1, 2, 3, 4), we achieved the best balance between the computational cost and the quality of the clustering among the existing methods.

**Table 2:** Computational cost of the similarity measures.

Method		Computation time (s)	
	Normal	Plus flanking regions	Plus unrelated sequences
proposed	95	222	199
FOLDALIGN	71748	226066	167228
LocARNA	9704	64679	30287
Stem kernel	61	179	138
LA kernel	71	163	160
CLUSTALW	4	43	6

The total time for computing similarity matrices is shown for three versions of the dataset.

In the design of the proposed method, our idea was to improve the reliability of approximate algorithms by the information of suboptimal solutions in their dynamic programming frameworks. Among LocARNA and FOLDALIGN, which only use *one optimal* solution in their approximate Sankoff-style algorithms, there was a trade-off that LocARNA was faster but less accurate than FOLDALIGN (Figure 1, and Table 2). Stem kernel, which utilizes a *subset of* solutions in the more approximate Sankoff-style algorithm, partly improved this problem, being faster and more accurate than LocARNA. Our method, which utilizes *all possible* solutions in the combination of the Smith-Waterman algorithm and the McCaskill algorithm, successfully overcome the trade-off. These results suggest that our strategy is essential to enable fast and accurate clustering of ncRNAs.

## Conclusions

We have described a new method for the hierarchical clustering of ncRNAs, which can be applied to the identification of novel ncRNA families. Our method can achieve the best balance between the computational cost and the quality of the clustering compared to the existing methods.

The performance of the clustering is determined by similarity measures based on the scores of structural alignment. The existing similarity measures, which only use *one optimal* structural alignment, suffer from the trade-off between time-consuming accurate algorithms and fast approximate algorithms. Our similarity measure, which is designed to utilize *all possible* sequence alignments and *all possible* secondary structures, have overcome this problem. The improvement is especially remarkable when the similarity among family members is not detectable at the primary sequence level.

In conclusion, our method enables fast and accurate clustering of ncRNAs, providing a promising way to explore the functional diversity of ncRNAs.

## Competing interests

The authors declare that they have no competing interests.

## Authors’ contributions

Y Saito and KS developed the algorithm and wrote the code. Y Saito performed the experiments and drafted the manuscript. Y Sakakibara initiated and coordinated the project. All authors have read and approved the final manuscript.
